# Effect of Anesthetics on Functional Connectivity of Developing Brain

**DOI:** 10.3389/fnhum.2022.853816

**Published:** 2022-03-11

**Authors:** Xu Chen, Xuemei Zheng, Jianghui Cai, Xiao Yang, Yonghong Lin, Mengjun Wu, Xiaofan Deng, Yong G. Peng

**Affiliations:** ^1^Department of Pharmacy, Chengdu Women’s and Children’s Central Hospital, School of Medicine, University of Electronic Science and Technology of China, Chengdu, China; ^2^School of Medicine, University of Electronic Science and Technology of China, Chengdu, China; ^3^Department of Obstetrics, Chengdu Women’s and Children’s Central Hospital, School of Medicine, University of Electronic Science and Technology of China, Chengdu, China; ^4^Department of Gynecology, Chengdu Women’s and Children’s Central Hospital, School of Medicine, University of Electronic Science and Technology of China, Chengdu, China; ^5^Department of Anesthesiology, Chengdu Women’s and Children’s Central Hospital, School of Medicine, University of Electronic Science and Technology of China, Chengdu, China; ^6^Center of Organ Transplantation, Sichuan Provincial People’s Hospital, Sichuan Academy of Medical Sciences, Chengdu, China; ^7^Department of Anesthesiology, College of Medicine, University of Florida, Gainesville, FL, United States

**Keywords:** anesthetic, neonatal, functional magnetic resonance imaging, neurotoxicity, functional connectivity, pediatric anesthesiology

## Abstract

The potential anesthetic neurotoxicity on the neonate is an important focus of research investigation in the field of pediatric anesthesiology. It is essential to understand how these anesthetics may affect the development and growth of neonatal immature and vulnerable brains. Functional magnetic resonance imaging (fMRI) has suggested that using anesthetics result in reduced functional connectivity may consider as core sequence for the neurotoxicity and neurodegenerative changes in the developed brain. Anesthetics either directly impact the primary structures and functions of the brain or indirectly alter the hemodynamic parameters that contribute to cerebral blood flow (CBF) in neonatal patients. We hypothesis that anesthetic agents may either decrease the brain functional connectivity in neonatal patients or animals, which was observed by fMRI. This review will summarize the effect and mechanism of anesthesia on the rapid growth and development infant and neonate brain with fMRI through functional connectivity. It is possible to provide the new mechanism of neuronal injury induced by anesthetics and objective imaging evidence in animal developing brain.

## Introduction

The anesthetics of causing neurotoxicity and neurodegenerative changes in the developing brain have been documented in animal studies over the past decades (Paule et al., 2011; Creeley et al., 2014; Rappaport et al., 2015; Andropoulos, 2018). A Centers for Disease Control and Prevention (CDC) survey shows: About 6 million children in the United States receive general anesthesia each year, that includes 1.5 million newborns and infants (DeFrances et al., 2007). Whether anesthetics can cause neurotoxicity in the development of infant’s central nervous system has become a research focus of social concern.

The potential anesthetic neurotoxicity on the neonate is an important focus of research investigation in the field of pediatric anesthesiology, particularly with prolonged or repeated exposures (Hansen et al., 2011; DiMaggio et al., 2012; Ing et al., 2012; Stratmann et al., 2014). Previous studies have explored the mechanisms of general anesthesia on the developing brains at the different pathways, receptors and molecular levels (Ho et al., 2017). However, there has no effective and intuitive monitoring modality that can directly reflect the effects of anesthetics on children’s brains, especially on neonate. Some studies have used functional magnetic resonance imaging (fMRI) to observe the structural and functional changes of the adult brain induced by anesthetics (Fox and Raichle, 2007), by measuring blood oxygen level-dependent (BOLD) signals and hemodynamics, which are indirect *in vivo* indicators of neural activity (Altman and Bernal, 2001). However, there have been lack of information on the neonates.

Functional magnetic resonance imaging (fMRI) has suggested that using anesthetics result in reducing functional connectivity may consider as core sequence for the neurotoxicity and neurodegenerative changes in the developed brain (Fox and Raichle, 2007). Anesthetics either directly impact the primary structures and functions of the brain or indirectly alter the hemodynamic parameters that contribute to cerebral blood flow (CBF) in neonatal patients. The anesthetic effect of propofol temporarily lessens the brain functional connectivity, that is, the connection network between the primary sensory cortex and the higher cortex is interrupted. Furthermore, fMRI has seen a surge in utilization as a diagnostic modality in neonatal patients (Andropoulos, 2018).

We hypothesis that anesthetic agents may either decrease the brain functional connectivity in neonatal patients or animals, which was observed by fMRI. This review will summarize the effect and mechanism of anesthesia on the rapid growth and development of infant and neonate brain with fMRI through functional connectivity.

## The Functional Connectivity Differences Between Newborns and Adult Brains

### Neurone

There are physiological differences between neonates and adult brains (McCann and Soriano, 2012; Fares et al., 2019; Zhang H. et al., 2019). In the initial development of the brain during early periods, neurons experience tremendous development, including maturation and differentiation. These biological processes establish a neuronal network for proper behavioral and cognitive function (Zhang H. et al., 2019). Meanwhile, synaptogenesis and the formation of numerous synaptic contacts, immature and redundant neurons are those that do not make synaptic connections. These neurons are fated to be pruned by apoptosis physiologically or being programmed to death. Neuroapoptosis is an ordinary part of the sequence of neuronal development when the brain is growing most rapidly (McCann and Soriano, 2012). The development of synaptogenesis mostly happens during the critical stages from the last trimester in pregnancy as a fetus to the first few years after birth (Mann and Kahana, 2015).

### Receptor

The major inhibitory γ-aminobutyrate (GABA) function is significantly different in immature and mature brains (Lei et al., 2012; Swanson and Maffei, 2019). GABA activates GABA receptors in adult brains, causing Cl^–^ inflow, cell membrane hyperpolarization, and inhibition of neuronic excitatory impulses (Lombardi et al., 2021). While in an immature brain, the low expression of transmembrane potassium-chloride cotransporter 2 (KCC2) causes the increase of Cl^–^ in nerve cells. GABAA receptor activation causes the Cl^–^ efflux, membrane depolarization, and excitatory impulse conduction are generated (Swanson and Maffei, 2019). Therefore, while GABA in mature animals plays the role as an inhibitory agent, it serves an excitotoxic agent in immature animals (Lei et al., 2012). Neonatal exposure to N-methyl-D-aspartate (NMDA) antagonists (e.g., ketamine, nitrous oxide), γ-aminobutyric acid (GABA) agonist drugs (e.g., propofol, barbiturates, benzodiazepines) leads to accelerated neurodegeneration (DeFrances et al., 2007).

### Metabolism

Glucose is the essential energy source for the adult brain, as the metabolism which it fuels up is extensive in adults. The neonatal brain, however, does not rely on glucose. Instead, ketone bodies are believed to be the energy supply for the neonate (Brekke et al., 2015). Due to the rapidly increasing energy demands for complex matriculation processes in cerebral structures and functions, the human brain’s energy source switches from anaerobic glycolysis, which fuels up the brain in the third trimester during pregnancy and the first several months after birth, to the more effective aerobic metabolism (Liu P. et al., 2014).

While neurons are activated, there accompanies an increase in regional blood volume and flow, as well as oxygen consumption. An increase in blood flow, a decrease in oxygen consumption, or deoxyhemoglobin concentration could result in a decrease in deoxyhemoglobin concentration. The T2*-weighted MRI can map T2*, the effective transverse relaxation time that can be increased by the deoxyhemoglobin concentration decrease in specific areas (Koopmans and Yacoub, 2019). The cerebral hemodynamics and oxidative metabolism, as a result, are being relied on by BOLD contrast.

When comparing neural stimulation in time slots and the rest, the BOLD signal is often found incremental when periods of neural stimulation happen, which is believed to indicate that the dominant variable in BOLD-based fMRI is the hemodynamic response (Kundu et al., 2017). The BOLD signal reflects “spontaneous neuronal activity” in the quiescent state and indicates the spontaneous discharge of neuronal groups that spread to other groups via the white matter axon connection, a large-scale, coordinated BOLD variation pattern, or form a functional connection (Chen et al., 2020). Besides, more frequent functional connectivity patterns inherit the structure of anatomical connectivity under anesthesia, with fewer small-world properties and lack negative correlations, linking the dynamics of whole-brain functional connectivity patterns and states of consciousness (Barttfeld et al., 2015; Ma et al., 2017).

### The Network and Functional Connectivity

Defined as the temporal dependency of patterns of neuron activation in brain regions that are anatomically separated (van den Heuvel and Hulshoff Pol, 2010), functional connectivity has been widely accepted in many different areas of clinical applications, including studies to evaluate the effects of anesthetics (Alkire, 2008; Boveroux et al., 2010; Deshpande et al., 2010; Wang et al., 2011, 2019; Palacios et al., 2013; Ranft et al., 2016; Blain-Moraes et al., 2017; Fortin et al., 2017; Palanca et al., 2017; Li et al., 2018; Liebe et al., 2018). There is an increase in the intrinsic functional connectivity before birth, reported by a study conducted by fetal fMRI chronologically with occipital, temporal, frontal, and parietal regions (Jakab et al., 2014). The brain’s functional subnetworks then grows in variations with age after birth.

At birth, the primary sensorimotor and auditory networks are matured already; the connectivity in visual areas are dramatically enhanced during the first 3 months postnatally; the functional connectivity strength in the attention and default-mode regions is observed to experience a rapid increase during the first 6 months in infancy; finally, there are significant changes in the executive control network between 9 and 12 months after birth (Gao et al., 2015; Jakab et al., 2015). There are rising interests in thalamocortical connectivity development, as functions of the thalamus are diversely associated with the transformation of the sensory information from the periphery to the cortex and higher-order cognitive functions (Cao et al., 2017b). The functional connectivity develops dramatically as humans grow up. New-born babies are already equipped with functional connectivity of thalamus-sensorimotor and thalamus-salience, but not until the 12 months of age would the specialized thalamus-medial visual and thalamus-default mode connectivity emerge (Cao et al., 2014, 2017a; Toulmin et al., 2015). Despite substantial reorganization of functional connectivity, several large-scale network properties appear to be preserved across development, indicating the functional brain networks are organized in manners (Power et al., 2010). In awake rats, the maturation timelines of brain circuits were heterogeneous and system-specific, functional connectivity tended to decrease in subcortical circuits, but increase in cortical circuits during development (Ma et al., 2018). Colonnese et al. (2008) explored the age-related parameters of BOLD signal from its apparent inception on postnatal day 13 to adulthood, and they found that the regional BOLD response in these animals undergoes a systematic decline in latency and growth in amplitude over this period, maturation of hemodynamic responses correlated with age-dependent increases in susceptibility to inhibition of carbonic anhydrase.

## Anesthesia Neurotoxicity on Metabolism in Developing Brain

The physiology of a neonate’s brain is different from an adult. The developing central nervous system is exquisitely sensitive to anesthetics (McCann and Soriano, 2012). There are two pathways for anesthetic agents to have an impact on neonatal patients, a direct influence on the brain’s primary structures and functions or indirectly change the hemodynamic parameters that are responsible for the cerebral blood flow (CBF). The cerebral blood flow brings in more oxygen, which increases metabolism in the brain (Slupe and Kirsch, 2018). In general, it is when the brain is growing the most rapidly that of maximal injury to cells may occur. No evidence is found in terms of the effects of anesthetics administration in human new-borns and young children on brain structure or neurocognitive function. While animal studies have described neuronal apoptosis and neurocognitive deficits after exposure to anesthetics or sedatives (Ho et al., 2017).

Ketamine-induced neuroapoptosis has already been found in rats, mice, and monkeys. In one experiment with rats, seven rats were given at least seven doses of 20–25 mg/kg ketamine on their postnatal day showed neurotoxicity. However, the other rats that received the same dose four or fewer times did not (Scallet et al., 2004). There is also increased neurodegeneration found in monkeys when they were given ketamine in the last trimester of pregnancy and the fifth day of life, but not on day 35 of life (Slikker et al., 2007).

Other research reported slightly different results on mice. Mice that received one dose of 25 mg/kg ketamine per day for ten consecutive days did not show any neurodegeneration symptoms. However, neurodegeneration symptoms were detected when combined with thiopental or propofol (Fredriksson et al., 2007). Exposure of 50–60 mg/kg propofol increases neuroapoptosis both in new-born and mature rats/mice (Cattano et al., 2008). Except for the abovementioned molecular targets for ketamine, there is another factor that has the ability to cause anesthetic properties, namely NMDA receptor. Early preclinical reports that the use of ketamine could increase the cerebral metabolic rate of oxygen (CMRO2) and small case series documenting elevated intracranial pressure (ICP) (Evans et al., 1971; Nelson et al., 1980; Grimm et al., 2015).

However, recent studies conducted in humans have controversial results. It is discovered that no increased ICP were detected if ketamine administration is combined with other anesthetic practices (Wang et al., 2014), which indicated that the minimal changes of CMRO2 in humans in great possibility have no affection on increased utilization. Instead, it is the production of increased metabolite supply (Långsjö et al., 2003).

Controversial results were found in preclinical data collected from animal models on neurodegeneration after benzodiazepine administration. Benzodiazepine administration appears to be species-specific. Young mice that received one dose of 5 mg/kg diazepam are reported to have increased neurodegeneration, but rats were not (Fredriksson et al., 2004). Increased neuroapoptosis were found in new-born rats when they received 5–10 mg/kg pentobarbital or 40–100 mg/kg phenobarbital, but the same result was not detected with thiopental doses of 5–25 mg/kg (Asimiadou et al., 2005). The abovementioned results supported the conclusion that there is a dose-dependent increase in neurodegeneration in small rodents after benzodiazepine were injected, and mice have a higher susceptibility than rats. Research indicated that the matched reductions of dose-dependent CBF and CMRO2 might be a result of benzodiazepines with acute administration (Matthew et al., 1995). The development of tolerance to the CBF reduction effects were found in an experiment in the examination of the association between the use of chronic benzodiazepine and CBF (Roy-Byrne et al., 1993). Although there are no casual effects established, however, the largest declination were found of regional CBF alternation in cerebral areas responsible for memory formation, attention, and arousal; and during the administration of benzodiazepines, there are correlations between these changes and different brain areas with clinical effects observed (Liang P. et al., 2012).

The hypothesis was made on their function to uncouple flow-metabolism of modern halogenated volatile anesthetics, for instance, isoflurane, sevoflurane, and desflurane (Slupe and Kirsch, 2018). Decreased dendritic numbers and synaptic density, especially concomitant exposure to midazolam and nitrous oxide, were found when subjects were exposed to subclinical doses of halothane with an extended time span (Uemura et al., 1985). Increased neuroapoptosis were found in new-born mice when exposed to sevoflurane for subclinical 2 h, and a 6 h exposure to sevoflurane may cause abnormal social and learning behaviors (Zhang et al., 2008). Nitrous oxide exposure has been shown to induce caspase-3 (an enzyme associated with apoptosis) (Hentzen et al., 2020) expression and increase the degree of neuroapoptosis in the infant mouse (Yon et al., 2005; Ma et al., 2007). Positive results were found in non-human primates as well as small animals in limiting injuries after induced ischemia in experiments when volatile anesthetics were used (Kitano et al., 2007).

Decreased critical CBF even lower than injuries after rising electroencephalogram (EEG) were found when isoflurane was in use during carotid endarterectomy (Michenfelder et al., 1987). Likewise, when desflurane is involved in temporary cerebral artery clipping, cerebral oxygenation would be preserved (Hoffman et al., 1998).

“Necroptosis” is a novel pathway of necrosis. It is reported that sevoflurane exposure elicited neurotoxicity within neonatal hippocampal neurons and tissues of rats, and blockade of both apoptosis and necroptosis alleviated sevoflurane-induced cognitive impairment (Xu et al., 2021). Pyroptosis is a novel type of programmed cell death with inflammation, playing an important role in the mechanism of multiple neurological diseases. Dai et al. (2021) indicated that repeated sevoflurane exposure may induce overreaction of pyroptosis with neuroinflammation and neurocognitive impairment in developing rats. Multiple studies have emphasized the importance of neuroinflammation caused by anesthetics exposure. Yang and Yuan (2018) found that anti-IL-17A can alleviate neuroinflammation and oxidative stress via inhibiting NF-κB pathway, thereby attenuating postoperative cognitive dysfunction (POCD) in aged rats anesthetized with sevoflurane. The mechanism of sevoflurane-induced neuroinflammation may involve microglial activation, blood-brain barrier breakdown, changes in gut microbiota, and ease of cholinergic neurotransmission (Huang et al., 2021).

Meanwhile, it has not been fully proved in specific life periods such as the neonate. Instead, toxicity is being found to attribute to volatile anesthetics only appearing in preclinical trials in this period (Davidson and Sun, 2018; Jevtovic-Todorovic, 2018). Consequently, without results from prospective randomized controlled trials, no affirmed conclusions should be made with insufficient evidence that volatile anesthetics plays an important role as neuroprotective or neurotoxic agents in primates, especially humans.

A study on mice in the examination of the relationship between propofol and neuronal structure as well as the neurocognitive performance found increases in neurodegeneration, behavioral, and learning impairment when propofol was applied, and the effect was identified with dependence on doses.

Neurological sequelae were not detected when neonate was exposed to 10 mg/kg propofol, however, increased neurodegeneration and disruption of spontaneous activity were found when 60 mg/kg of propofol by itself or 10 mg/kg of propofol and 25 mg/kg of ketamine were applied (Fredriksson et al., 2007). Animal studies using *in vitro* preparations demonstrate clinical doses of propofol-induced neuronal cell death, while not enough evidence is deployed in immature animals to justify the existence of injurious effects of propofol on neuronal survival, arborization, and electrophysiological function (Spahr-Schopfer et al., 2000; Engelhard et al., 2004).

Propofol is proved to cause CBF reduction and CMRO2 decrease dramatically, compared with Bispectral Index (BIS)-equivalent delivered volatile anesthetics at equipotent doses (Schlünzen et al., 2012). The mechanism that CBF reduction appears to be considered to arise from the entire flow-oxidative metabolism coupled. When propofol is injected into vessels directly *in vitro* preparations, vasodilation can be found in this process (Park et al., 1992).

## Functional Magnetic Resonance Imaging Recording Effect of Different Anesthetics on Functional Connectivity

A series of changes in brain functional network connections are closely related to cognitive functions such as memory retrieval, coding and consciousness (Betzel et al., 2014). Anatomically, the brain is divided into regions that function differently. Brain connections include structural and functional connections. Structural connections are the connections of nerve fiber bundles and other substances, while functional connections are the connections obtained by measuring the activity of various brain regions by some monitor technologies (Poirel et al., 2011; Park and Friston, 2013). The BOLD signal showed spontaneous fluctuations associated with time patterns of neural network activity at rest (Li et al., 2020). The correlation of spontaneous signal fluctuations between discrete regions is called functional connectivity and is believed to be the basis of communication within the brain network. Interestingly, Liang Z. et al. (2012) reported that the long-distance connections were not preferentially reduced in the anesthetized condition, arguing against the hypothesis that loss of long-distance connections is characteristic to unconsciousness. Currently, fMRI techniques are commonly used to capture the information flow between brain regions to analyze the functional connectivity due to its non-invasive capacity, allowing for the direct comparison of large-scale murine and human brain functions (Jiménez et al., 2020).

The application of fMRI opens an avenue for bidirectional translational strategies to address fundamental questions ranging from neurological disorders to the nature of consciousness (Reimann and Niendorf, 2020).

### Propofol

Intravenous anesthetic propofol is the most commonly used induction and maintenance drug in clinical anesthesia, which mainly plays the role of general anesthesia by activating the GABA receptor in the locus ceruleus (Walsh, 2018). There is a hypothesis that the loss of consciousness induced by general anesthetics during surgery may be related to the disturbance of synaptic function by anesthetic drugs at present, which impairs the coordination of cortical activity (Alkire, 2008). The coordination of cortical activity depends not only on the anatomical connections between brain regions, but also on the functional connections between brain regions. Many studies have found that the brain spontaneously produces obvious fluctuation signals during deep sedation with propofol, which will cause changes in multiple brain network connections (Boveroux et al., 2010). It may represent a decrease in consciousness dependent on changes in cerebral blood flow (CBF). The anesthetic effect of propofol temporarily reduces the brain functional structure, that is, the connection network between the primary sensory cortex and the higher cortex is interrupted (Gross et al., 2019). Blain-Moraes et al. (2017) found that the global network efficiency of patients under general anesthesia decreased unconsciously and returned to the baseline level during the recovery period. Li et al. (2018) divided 24 healthy subjects into sleep group and propofol sedation group, the results showed that during sleep, regional homogeneity (ReHo) was mainly weakened in the frontal lobe, but enhanced in the brainstem. During propofol sedation, ReHo changed in various brain regions, including cingulate gyrus, thalamus and cerebellum, and the functional connections between cortex and subcortex were significantly weakened. With the deepening of sedation, cerebellar-based functional connections were weakened. However, those based on thalamus and brainstem were enhanced. This study shows that sleep mainly depends on brainstem and frontal lobe function while sedation tends to affect a wide range of brain functional network connections (Li et al., 2018). A large number of studies have confirmed that propofol temporarily may impair the functional structure of the brain to some extent and blocks the signal connection between the sensory cortex and the frontal parietal-cortex (Adapa et al., 2014; Liu X. et al., 2014; Song and Yu, 2015; Liu et al., 2017). However, it is important to identify the effect of anesthetics on the fMRI signal quality. For instance, after HRF attenuating, it limits the statistic power to detect functional activity, leading to the smaller fluctuations of fMRI signal; besides, fMRI signal suffers from low temporal signal-to-noise ratio, making it even more difficult to get detectable functional activity under anesthesia.

### Inhalation Anesthetics

Sevoflurane has been widely used in clinics in recent years. However, the specific mechanism of sevoflurane on the central nervous system is not clear. Some studies have found that the functional networks of different brain regions have different effects after inhaling different concentrations of sevoflurane (Deshpande et al., 2010; Palanca et al., 2017). The fMRI results showed that when healthy volunteers inhaled 2 and 1% sevoflurane, respectively, and the brain network correlation decreased in both groups. However, the brain network correlation decreased more significantly in 2% sevoflurane group than in 1% group, especially the network connection information of anterior and posterior cingulate gyrus and secondary parietal cortex. This shows that sevoflurane has an extensive inhibitory effect on the cerebral cortex under shallow anesthesia, resulting in a decrease in the local connectivity of the frontal cortex (Walsh, 2018). This is similar to the results of Ranft et al. (2016) that sevoflurane reduces the connection between the frontal lobe and the thalamic cortex. Wang et al. (2011) found that inhaling different concentrations of isoflurane can lead to spontaneous blood oxygen level-dependent signal fluctuations in somatosensory and motor areas of mice. Masamoto et al. (2009) investigated the dose-dependent effects of isoflurane on neurovascular coupling in the rat somatosensory cortex, and found that field potential (FP) response to single-pulse stimulation remained unaffected across the different levels of isoflurane (1.1–2.1%), whereas the CBF response to single-pulse stimulation increased dose-dependently. Brynildsen et al. (2017) used a low dose of dexmedetomidine in combination with a low dose of isoflurane in rats, systematically characterizing physiological conditions for fMRI experiments under this anesthetic regimen; they found both evoked BOLD response and resting-state fMRI signal remained stable during the 90–150 min time window, while autonomic physiological parameters maintained near-normal conditions during this period. Interestingly, Zhang Z. et al. (2019) reported that isoflurane-induced burst suppression increases intrinsic functional connectivity of the monkey brain. Another study found that distinct resting-state networks covering functionally specific sub-regions of the sensorimotor system were observed under light anesthesia with 1.0% isoflurane; however, they gradually merged into a highly synchronized and spatially less-specific network under deep anesthesia with 1.8% isoflurane (Liu et al., 2013). Golkowski et al. (2019) analyzed the fMRI data obtained by volunteers who received propofol (*n* = 16) and sevoflurane (*n* = 14) during general anesthesia. The results showed that the functional connections of the brain in the thalamus and hippocampus changed significantly during general anesthesia (Golkowski et al., 2019). During general anesthesia, the activity in the brain network decreased and the network connectivity decreased.

### Ketamine

Ketamine is a non-specific antagonist of the NMDA receptor, which mainly stimulates the limbic system of the thalamus and blocks the signal transduction of the reticular tract of the spinal cord, thus blocking the ascending of the stimulation signal. Ketamine is a general anesthetic with a definite analgesic effect (McCann and Soriano, 2019; Abdollahpour et al., 2020). A fMRI study on ketamine found that signals in sensory cortex, motor cortex, thalamus and cingulate gyrus were suppressed after ketamine anesthesia (Fortin et al., 2017). Liebe et al. (2018) used fMRI to study the resting-state functional connection locus ceruleus (LC) before and 1 h after subanesthetic ketamine (0.5 mg/kg) injection. The results showed that the drug was related to time, and the resting state functional connection between locus ceruleus and thalamus decreased compared with the baseline level after ketamine administration (Liebe et al., 2018). At present, some fMRI studies have reported abnormal functional connections in the frontal lobe and cingulate gyrus of long-term heroin users and smokers (Liebe et al., 2009). Rao et al. (2017) also used fMRI technique to explore the effect of ketamine on brain functional connectivity in monkeys. It was confirmed that after a single dose of ketamine was injected into anesthetized monkeys, the local functional connectivity signals in caudate nucleus and hippocampus increased. However, the functional activity of prefrontal cortex decreased (Song and Yu, 2015).

## Discussion on Mechanism

The potential neurotoxicity of anesthetics in neonates and young children is one of the most pressing issues facing the field of pediatric anesthesia. Several studies based on animals have shown that general anesthetics can cause neurotoxicity effects during brain development, affect synaptic plasticity, and induce apoptosis, and finally affect learning and memory function in adulthood (Yang et al., 2020). The rapid development of the fetal brain may make this patient population most vulnerable to the neurotoxicity of anesthesia. Previous researchers have studied the mechanism of general anesthesia from the ion channels, receptors and molecular levels. With the transformation from molecular mechanism to neural network of general anesthetics research, some studies have found that endogenous sleep-awakening system, promoting awakening, consciousness maintenance, anesthesia recovery and other related network nuclei are involved (Fu et al., 2017). However, how does general anesthetics act on brain functional areas and the specific mechanisms of induction and maintenance of anesthesia remain unclear. It is hypothesized that the loss of consciousness induced by anesthetics is due to the fact that anesthetics block or inhibit the functional connection of brain tissue, resulting in the loss of the brain’s ability to integrate, process and analyze information, and at the same time, the body’s brain functions such as memory, consciousness and cognition are impaired (Masamoto and Kanno, 2012). Till now, some anti-oxidant drugs, dexmedetomidine, as well as a rich living environment and exercise have been proven to reduce the neurotoxicity of anesthetics (Yang et al., 2020).

**FIGURE 1:**
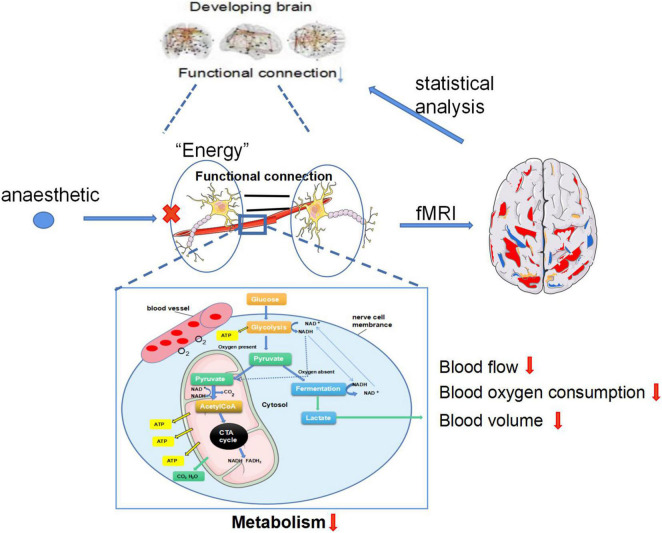
The speculated mechanism of fMRI recording the anesthetics alter functional connections in the developing brain. (1) Neurons operate autonomically in resting state, the brain provides energy for neurons through glycolysis. Images of resting state brain can be obtained through fMRI and functional connections of resting state brain can be obtained through statistical analysis. (2) Anesthesia exposure can lead to decreased the blood flow, oxygen consumption and blood volume of neurons, which affects the glycolysis process of the brain, resulting in decreased energy of neurons, reduced BOLD signal captured by fMRI and decreased functional connectivity.

Numerous fMRI studies have shown that anesthetics alter functional connections in the brain, but only in animals and in adults. The speculated mechanism is shown in Figure 1. Does it happen in neonates or children? At present, no studies have been conducted on the changes in brain structure and function induced by anesthesia in neonates or children. According to this review, we know that the physiological functional connection of newborn brain differs from adult brain, and many animal studies have determined that anesthetics are indeed neurotoxic to the developing brain. The human brain develops rapidly during the neonatal period, the brain is more sensitive to anesthetics at this very period. We hypothesized that if anesthetics can change the structure and function of the network in adults, they may also affect the newborn brain.

Functional magnetic resonance imaging studies can indicate the parallelism and connection patterns of brain neural networks and the characteristics of interconnections between different brain regions. It can provide dynamic and functional information of brain microstructure by reflecting the level of brain neural activity. fMRI brain functional imaging provides a new way to study the action target of anesthetic drugs and the mechanism of analgesia. The related research on brain functional connections has developed rapidly in recent years. fMRI can not only perform real-time functional imaging of brain functional activities or hemodynamic changes in specific regions, but also obtain the structural and functional image features of various brain regions at the same time. It has many advantages in analyzing the functional connections of brain regions under different conditions, which provides an important reference for the diagnosis and treatment of clinical brain neurological diseases. Comprehensive molecular, electrophysiological, metabolic, vascular and network imaging studies will cover interactions from the molecular level to the distribution of regional networks in the brain. fMRI technique is of practical value in exploring the effects of general anesthetics on brain functional connections, and can comprehensively and accurately understand the structural and functional changes of brain tissue. It is expected that the further development of this technology and method will explore the action mechanism of anesthetics in various brain regions, and reduce the occurrence of anesthesia-related complications.

It intends to provide more accurate suggestions for the formulation of individualized anesthesia programs for patients with different diseases and complications, and to guide anesthesiologists to use narcotic drugs in neonate more safely and rationally.

## Conclusion

Neural activation is accompanied by metabolism by a regional increase in blood flow, blood volume and oxygen consumption, the BOLD-fMRI could differentiate the changes in both cerebral hemodynamics and oxidative metabolism. Decreased functional connectivity may be the core sequence for the neurotoxicity and neurodegenerative changes after administration anesthetics in the developing brain, and then fMRI techniques could capture the information flow between brain regions to analyze the functional connectivity of neonates. It is possible to provide the new mechanism of neuronal injury induced by anesthetics and objective imaging evidence in animal developing brain.

## Author Contributions

MW, XD, and YP contributed to the conception of the review. JC searched the information about the review. XC and XZ contributed significantly to analysis and manuscript preparation. XC performed the data analyses and wrote the manuscript. XY and YL helped to perform the analysis with constructive discussions. All authors contributed to the article and approved the submitted version.

## Conflict of Interest

The authors declare that the research was conducted in the absence of any commercial or financial relationships that could be construed as a potential conflict of interest.

## Publisher’s Note

All claims expressed in this article are solely those of the authors and do not necessarily represent those of their affiliated organizations, or those of the publisher, the editors and the reviewers. Any product that may be evaluated in this article, or claim that may be made by its manufacturer, is not guaranteed or endorsed by the publisher.
